# Three‐liquid‐phase salting‐out extraction of eicosapentaenoic acid (EPA) and docosahexaenoic acid (DHA)‐rich oils from *Euphausia superba*

**DOI:** 10.1002/elsc.202000098

**Published:** 2021-07-26

**Authors:** Liaqat Zeb, Xin‐Nan Teng, Muhammad Shafiq, Shu‐Chang Wang, Zhi‐Long Xiu, Zhi‐Guo Su

**Affiliations:** ^1^ School of Bioengineering Dalian University of Technology Dalian P. R. China; ^2^ State Key Laboratory of Biochemical Engineering Institute of Process Engineering Chinese Academy of Sciences Beijing P. R. China

**Keywords:** Antarctic krill, docosahexaenoic acid, eicosapentaenoic acid, extraction efficiency, fluoride, three‐liquid‐phase salting‐out extraction system

## Abstract

The TLPSOES parameters were optimized by response surface methodology using Box–Behnken design, which were 16.5% w/w of ammonium citrate, 17.5% w/w of ethanol, and 46% w/w of n‐hexane at 70 min of stirring time. Under optimized conditions the extraction efficiency attained was 90.91 ± 0.97% of EPA, 90.02 ± 1.04% of DHA, and 91.85 ± 1.11% of KO in the top n‐hexane phase. The highest extraction efficiency of proteins and flavonoids, i.e. 88.34 ± 1.35% and 79.67 ± 1.13%, was recorded in the solid interface and ethanol phase, respectively. The KO extracted by TLPSOES system consisted of lowest fluoride level compared to the conventional method and whole wet krill biomass. The TLPSOES is a potential candidate for nutraceutical industry of KO extraction from wet krill biomass.

## Graphical abstract

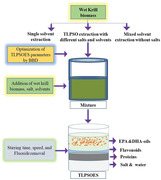

**Abbreviations**
DHA: docosahexaenoic acid
EPA: eicosapentaenoic acid
KO: krill oils
TLPSOES: three‐liquid‐phase salting‐out extraction system

## 1 INTRODUCTION

*Euphausia superba* (Antarctic krill) is a prominent crustacean species living in the regions of Polar Antarctic Oceans, which has been estimated to reach up to 379 million tons [1]. Recently, Antarctic krill is getting more attention worldwide because of its nutritional and functional potentials [2]. Antarctic krill comprises of abundant valuable nutrients like proteins, oils, and various smaller bioactive ingredients, such as vitamin A, astaxanthin, flavonoids, tocopherols, and minerals [2, 3]. The whole krill contains 11.9–15.4% proteins, which have higher quality than milk proteins [4, 5]. Interestingly, a novel flavonoid component in krill was characterized with a similar structure to 6, 8‐di‐C‐glucosyl luteolin [2, 6–8]. It was reported to evidence its effects on skin prevention from ultraviolet B (UVB) radiation at a low concentration of 7 mg/100 mL [2]. Furthermore, natural flavonoids can eliminate the risk of asthma, heart disease, cancer, and protect the brain [9]. More importantly, nutritional and positive health‐related effects of krill oil (KO) have been documented, including neuroprotection, anti‐obesity, cardiovascular disease (CVD) prevention, anti‐diabetic, anticancer effects, and anti‐inflammatory activities [2]. KO was approved or authorized as GRAS (Generally Recognized as Safe) or valuable food component by the U.S. Food and Drug Administration (FDA), European Community, and Chinese Government in 2008, 2009, and 2014, respectively.

It has been reported that krill is composed of 0.5–3.6% lipids [10] with diverse classes [1, 11]. These diverse lipids classes are mainly characterized by phospholipids (PLs) integrated with docosahexaenoic acid (DHA, C22:6, n‐3) and eicosapentaenoic acid (EPA, C20:5, n‐3) [1, 3]. As reported previously, n‐3‐PUFAs‐integrated PLs are more proficiently incorporated and absorbed into cell membranes than triglycerides (TAGs) [11, 12]. The efficient incorporation of KO into the cell membrane makes it a better choice than fish oil [12]. The EPA and DHA are omega‐3 polyunsaturated fatty acids which are the important component of the human cerebral cortex, skin, and brain [13, 14, 15]. Moreover, EPA and DHA have been documented for nutritional and health‐related applications [16]. However, most of krill is consumed for the aquaculture feeds due to the maximum astaxanthin amount, but only approximately 12% of krill is utilized by mankind [4, 5]. The main limit factor is a high concentration of fluoride in Antarctic krill, such as 570 ppm fluorides found in muscles, 2594 ppm in the integument, and 6 ppm in the soft tissues [5, 17]. The high fluoride level in water can lead to mottling of the human teeth up to 30–50% [4]. Besides, chronic fluorosis may cause osteosclerosis, bony exostoses, calcification of ligaments and tendons, and renal calculi [4, 5]. Therefore, the fluoride level in foods is limited strictly by the governments.

The KO has been documented to be extracted from various krill biomasses, including fresh krill and dried krill powder [2]. The extraction of KO from dry krill powder will consume more energy than direct extraction from fresh krill [18]. Therefore, various direct extraction approaches have been applied to isolate the KO, including supercritical fluid extraction, enzyme‐assisted pretreatment extraction, non‐solvent extraction, and solvent extraction [2, 12, 18]. Supercritical CO_2_ has lower solubility for polar lipids (PLs) [12], while conventional extractants (e.g. methanol and chloroform) are toxic and need addition in large amounts. Moreover, all these extraction technologies focused prominently on KO quality and yield, but no simultaneous extraction and separation of KO and other byproducts in a single unit operation. For example, the extracted KO with high fluoride level using solvent extraction still needed further purification to reduce the fluoride level, e.g. adsorption of activated clay, including stirring for 120 min, centrifugation, and evaporation of the solvent [17]. In fact, the extraction of KO with optimum fluoride directly from wet krill biomass is still a challenge for its commercialization. Although the krill is a rich source of nutritional components, rare methods are reported to extract and separate all these components in a single system with high product yield.

Three‐liquid‐phase salting‐out extraction system (TLPSOES) composed of organic solvents and salts has been documented to separate various ingredients from biomass in a single system. It was utilized to recover proteins, saponins, polysaccharides, and oils from processing sea cucumber [19]. Moreover, steroidal diosgenin and saponins were separated from *Dioscorea zingibernsis* by a TLPSOES [20]. Also, a microwave‐assisted TLPSOES was employed to obtain DHA‐rich oils, proteins, and polysaccharides from microalgae *Schizochytrium limacinium* SR21 [15]. More importantly, various valuable ingredients will be distributed in different phases with high yields in a TLPSOES according to their polarity and solubility [20, 21, 22, 23]. It is worth noting that TLPSOES has multiple benefits compared to conventional solvent extraction, such as simple process, high product yield, and saving energy [20, 21].

To the best of our knowledge, no literature studies have been reported regarding TLPSOES for wet *Euphausia superba*. Therefore, in this study, a basic novel TLPSOES was investigated to separate multiple components from wet *Euphausia superba* biomass. The EPA & DHA‐rich oil was simply purified from high fluoride levels in TLPSOES compared to conventionally extracted oil. Based on response surface methodology (RSM), the Box–Behnken design (BBD) was used to optimize the parameters of TLPSOES.

**PRACTICAL APPLICATION**

Antarctic krill (*Euphausia superba*) is getting attention worldwide because of its abundant standing stock, nutritional and functional potentials resource for human use. However, the utilization of Antarctic krill and krill oils (KO) is restricted due to high fluoride content issues. A novel three‐liquid‐phase salting‐out extraction system (TLPSOES) was developed to extract eicosapentaenoic acid (EPA) and docosahexaenoic acid (DHA)‐rich oils with lowest fluoride level, as well as abundant proteins, and flavonoids from wet krill biomass in the same system.

## 2 MATERIALS AND METHODS

### 2.1 Materials

Antarctic krill was provided by the Liaoning Province Dalian Ocean Fishery Group Corporation, and stored at −70°C in a refrigerator. The Antarctic krill biomass was composed of total amounts of proteins (13.95 ± 1.24%), EPA (12.47 ± 1.69%), DHA (8.62 ± 1.28%), oils (2.87 ± 1.43%), and flavonoids (0.004 ± 0.002%) w/w, respectively. The total amount of EPA and DHA in oil was determined according to Zeb et al. [24], while the total oil according to Bligh & Dyer's [25]. The total amount of protein was analyzed using Kjeldahl method (GB5009.5‐85). The total flavonoid was determined by a modified method according to Zeb et al. [24]. Each experiment was repeated until the highest yield was achieved. Standard chemicals such as EPA, DHA, heptadecanoic acid, quercetin, and bovine serum albumin (BSA) were purchased from Sigma‐Aldrich (purity greater than 99%). Sodium hydroxide, fluoride, hydrogen peroxide, sodium carbonate, ammonium citrate, ammonium sulphate, sodium sulphate, sodium citrate, potassium sulphate, and all solvents were purchased from Sinopharm Chemical Reagent Co., Ltd. All other chemicals were of analytical grade. At the same time, deionized water was used for solution preparation.

### 2.2 Extraction of KO

#### 2.2.1 Extraction of KO by conventional solvent methods

Extraction of KO was conducted using single or mixed solvents without the addition of salt. The n‐hexane and n‐butanol were used as a single solvent, and a mixture of ethanol and n‐hexane as a mixed solvent. A certain gram of krill biomass was taken and then added to the above solvent. Further, all the samples were stirred at 200 rpm for 120 min at 40°C. In the end, samples were let to separate phases or centrifuged at 3700 rpm for 7 min. Finally, the KO was obtained using a rotary evaporator.

#### 2.2.2 Extraction of KO by TLPSOES

According to literature studies, we chose different salts (ammonium citrate, ammonium sulphate, sodium sulphate, potassium sulphate, and sodium citrate) and solvents (propanol, n‐butanol, acetone, ethanol, and n‐hexane) to design TLPSOESs. Notably, the effects of salts and solvents were investigated to separate EPA & DHA‐rich oils with low fluoride level from wet krill biomass. The TLPSOES composition range of solvents and salts was from 5 to 60% w/w, respectively. Besides, the TLPSOE procedure was repeated with changes in the addition of salts and solvents. All the TLPSOES samples were kept on stirring at 200 rpm for 150 min at 37°C. In the end, samples were let to separate phases or centrifuged at 3700 rpm for 7 min. The TLPSOES parameters were further identified and optimized using response surface methodology.

A TLPSOES was set to be optimized with multiple parameters, which was composed of ammonium citrate, ethanol, n‐hexane, water, and krill biomass. Various amounts of ammonium citrate, water and wet krill were added into a tube and vortexed for 3–4 min. Further different concentrations of ethanol and n‐hexane were added into the tubes, then stirring at 200 rpm for 70 min at 37°C. The sample was taken out and let to separate phases. Within 5 min the different transparent phases or the samples can also be centrifuged at 3700 rpm for 7 min. The four phases were formed including top n‐hexane‐phase, middle ethanol‐phase, bottom aqueous phase, and solid interface between the middle and bottom phase.

The Box–Behnken design (BBD, by Minitab‐19 software) was generated using response surface methodology (RSM) to study key independent variables, namely ammonium citrate (A), stirring time (B), ethanol (C), and n‐hexane (D). The three‐level BBD was spherical with six central points to attain the highest extraction efficiency of responses KO, EPA, DHA, proteins and flavonoids. Table 1 showed the range value of the independent variables. All the experiments were performed in randomized order, while Analysis of Variance (ANOVA) was used to analyze the responses statistically.

**TABLE 1:** The data ranges of significant variables used for Box–Behnken Design (BBD)

No.	Variables	Low‐value	High‐value
A	Ammonium citrate (%, w/w)	10	30
B	Stirring time (min)	30	130
C	Ethanol (%, w/w)	10	25
D	n‐Hexane (%, w/w)	25	60

The extraction efficiency (%) for all components was calculated using the following equation.

$$\mathrm{Extraction}\;\mathrm{efficiency}\;\left(\%\right)=\frac{M1}{M2\;\;\times \;A}\;\times \;100\%$$

where *M*_1_ (g) is the recovered KO or other components weight in TLPSOES, *M*_2_ (g) is the initial krill material biomass used, and A (g/100 g) is the total oil content or other components obtained by standard method.

The partition coefficient of EPA and DHA was calculated using the following equation.

$$K=\frac{C_{t}}{C_{m}}$$

Wwhere *K* represent the partition coefficient, *C*_t_ concentration of EPA and DHA in the top phase (n‐hexane phase) and *C*_m_ concentration of EPA and DHA in the middle phase (ethanol phase).

The yield was calculated mass obtained on total mass in the sample.

### 2.3 Determination of fluoride contents

The samples as whole wet krill or extracted KO need to be digested before fluoride analysis. For whole wet krill, certain amounts of samples were taken into different tubes, and followed the experimental procedure to digest it. In each tube 1 mL Na_2_CO_3_ (with a concentration of 1 mol/L), 2 mL of NaOH solution (20 mol/L), and 3 mL H_2_O_2_ solution (concentration in mass 30%) were added in a series. Furthermore, the mixture was shaken using a vortex and digested at 100°C for 4 h. After digestion of the mixture at 100°C for 4 h, the sample was diluted up to 50 mL by deionized water. Finally, the samples were centrifuged at 5000 rpm for 5 min. The clear phase was collected, filtered through a 0.2 μm filter, and stored at 4°C in a refrigerator for further use. The extracted KO using TLPSOES or conventional extraction was treated according to the above procedure.

The conventional extraction of KO was carried out according to Bligh & Dyer method [25], by which chloroform and methanol were used as extractant. One gram of KO extracted by conventional method was dissolved in 40 mL of n‐hexane solvent. Then a certain gram of calcium chloride and calcium oxide was added as an absorbent agent. The mixture was stirred at 37°C for 150 min. Finally, the mixture was centrifuged at 5000 rpm for 5 min. The n‐hexane phase was collected and removed using rotary evaporation to attain KO.

The digested and filtered samples were further investigated for fluoride analysis by Ion Chromatography (IC). All the samples analyses were carried out on an IC DIONEX ICS‐5000 system connected with a conductivity detector (CD). The solution of potassium hydroxide was used as an eluent at 5 mM, and the flow rate was 1 mL/min. Samples were separated on column IonPac AS11‐HC (Diameter 4 × 250 mm), and the column temperature was 35°C. The suppressor ADRS‐4 mm was connected between the detector and column. The suppression current was 13 mA and injection volume 25 μL. The standard solution of fluoride was used to plot a calibration curve.

### 2.4 Analysis of bioactive components in TLPSOES

#### 2.4.1 Analysis of KO, EPA and DHA

KO in the top n‐hexane‐phase of a TLPSOES was measured by gravimetric method after removal of n‐hexane using rotary evaporator. The EPA and DHA contents were quantified using a Gas‐Chromatograph (GC) system after methylation of KO sample (about 20 mg) according to our previously reported method [15]. The fatty acids methyl esters (FAMEs) obtained were collected in n‐hexane solvent for GC quantification. The GC was fixed with an FID detector, and PEG‐20w capillary column (30 m × 0.25 mm, 0.1 μm film thickness). The temperature of oven was kept at 260°C, while the column temperature raised from 130–170°C for 1–10°C/min, and then increased to 210°C at 2.8°C/min. The internal standard heptadecanoic acid was used to quantify the EPA and DHA in the oil samples.

#### 2.4.2 Analysis of flavonoids and proteins

The middle phase of TLPSOES was cautiously separated, collected, diluted, and filtered for flavonoids analysis according to a modified method [24]. The sample absorbance was recorded at 420 nm in a spectrophotometer using quercetin as a standard flavonoid.

The crude proteins in the solid interface and middle phases of TLPSOES were quantified using Kjeldahl method (GB5009.5‐85) and Bradford assay [26], respectively. The experimental procedure for sample preparation was followed accordingly to Zeb et al., 2019 [15].

### 2.5 Statistical analysis

The experimental results were attained in triplicate (n = 3) and specified as means ± standard deviation. All the experimental data were analyzed using Microsoft Excel 2013, Minitab 19, and Origin 8.5 software. The experimental data were analyzed by ANOVA statistical software, while the least significant difference in one‐way ANOVA was used for multiple comparisons between groups. The *P* < 0.05 for experimental data meant significant difference, otherwise, insignificant difference [27].

## 3 RESULTS AND DISCUSSION

### 3.1 Extraction of KO from wet krill

**FIGURE 1:**
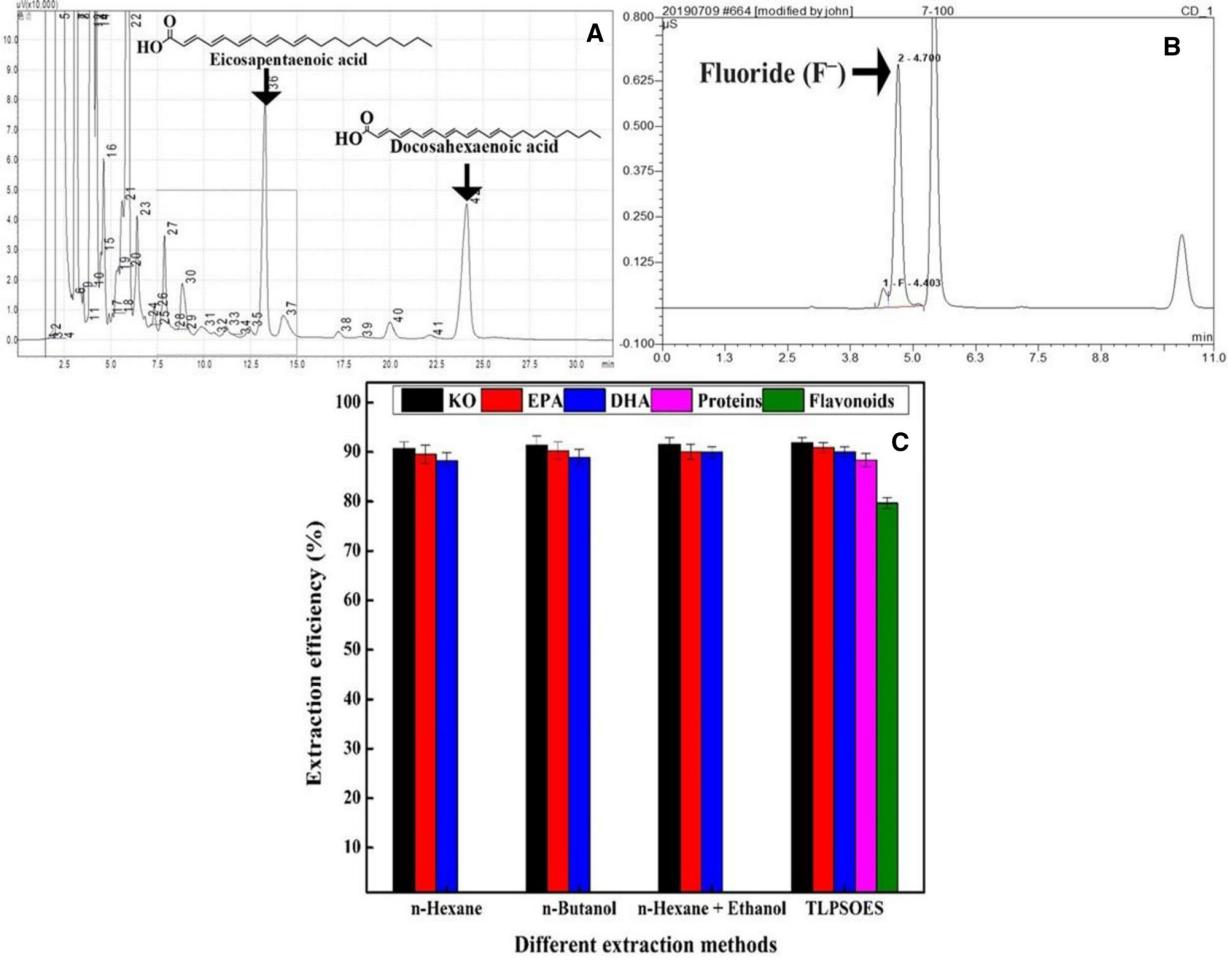
(A) The peaks positions of EPA and DHA identified by gas chromatography (GC). (B) The peak position of fluoride identified by ion chromatography (IC). (C) The comparison of conventional solvents extraction methods with TLPSOES

Various conventional extractions of KO from wet krill biomass were performed to investigate effects of solvents and extraction time on extraction efficiency. Both EPA and DHA (Figure 1A) were chosen as indicative components for the evaluation of different extraction methods. As indicated in Figure 1C, the extraction efficiency of KO, EPA, and DHA ranged from 88.22% to 91.85% among various extraction approaches. The extraction efficiency of various methods increased in the following order: n‐hexane (90.67%) < n‐butanol (91.34%) < n‐hexane & ethanol without salt (91.55%) < TLPSOES (91.85%) for KO, n‐hexane (89.56%) < n‐hexane & ethanol without salt (90.05%) < n‐butanol (90.23%) < TLPSOES (90.91%) for EPA. While the maximum extraction efficiency of DHA was 90.02% in case of TLPSOES. Compared with conventional solvent extraction without salt, e.g. KO extraction efficiency of 90.67% or 91.34% using n‐hexane or n‐butanol as solvent for 120 min, the TLPSOES showed the higher extraction efficiency of multiple components, i.e. 91.85 ± 1.11%, 90.91 ± 0.97%, 90.02 ± 1.04%, 88.34 ± 1.35%, and 79.67 ± 1.13% of KO, EPA, DHA, proteins, and flavonoids, respectively, in less extraction time (70 min). This is consisted with our previous reports for wet microalgae biomass and sea processing cucumber [15, 19], because n‐hexane in TLPSOES is favored for lipids extraction [28, 29, 30]. At the same time, salting‐out effect forces proteins and flavonoids to partition into the middle phase or the interface between the middle and bottom phase due to addition of salt in TLPSOES, as well as water into the bottom phase. For example, the extraction efficiency of KO, EPA, DHA, proteins, and flavonoids were 91.85 ± 1.11, 90.91 ± 0.97, 90.02 ± 1.04, 88.34 ± 1.35, and 79.67 ± 1.13%, respectively, using a TLPSOES composed of n‐hexane, ethanol, wet krill biomass, and ammonium citrate.

### 3.2 Selection of suitable TLPSOES

#### 3.2.1 Salts

**FIGURE 2:**
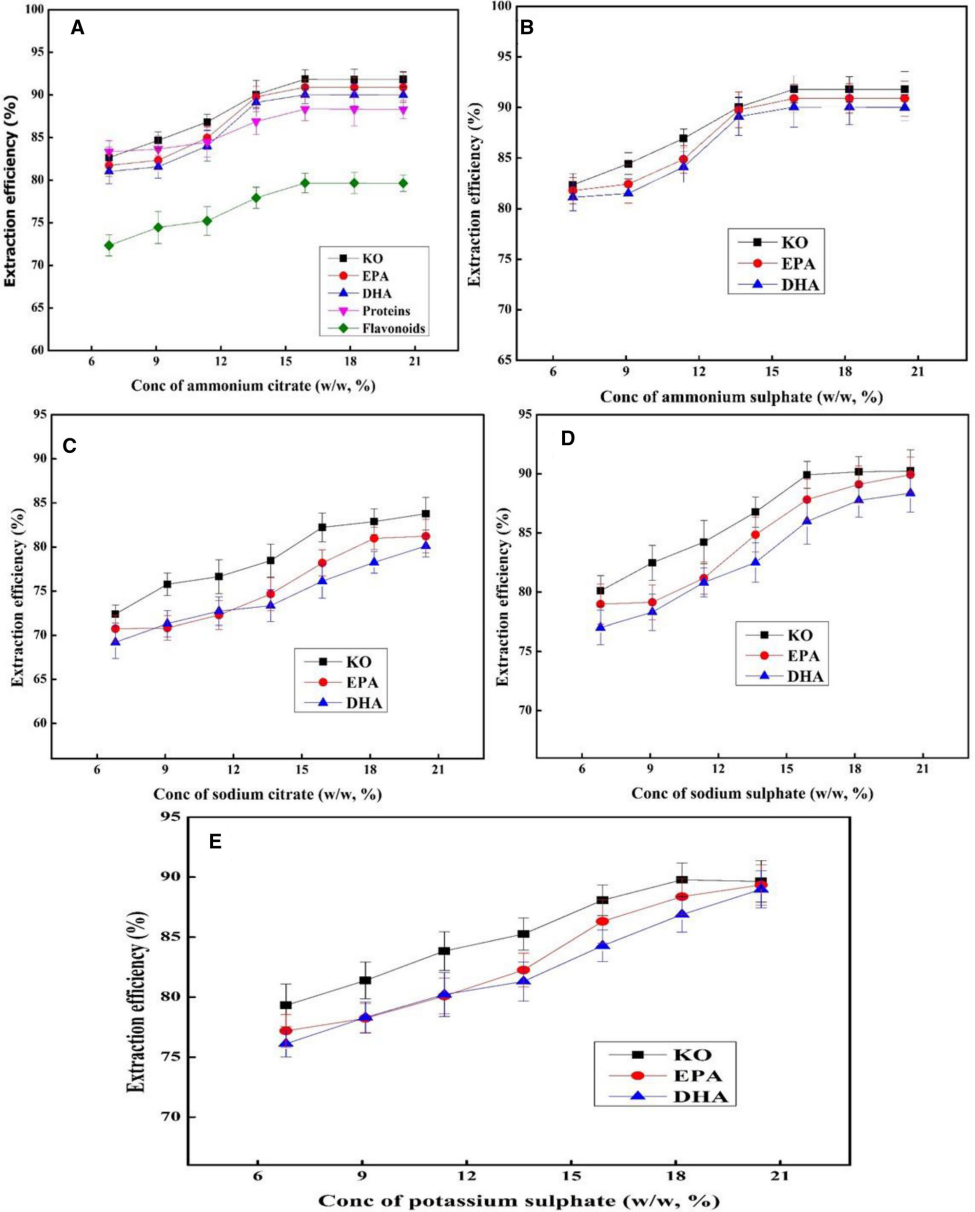
The influence of various salts on the extraction efficiency of KO, EPA, DHA, proteins and flavonoids in different TLPSOES using wet krill biomass. The salts consist of (A) ammonium citrate, (B) ammonium sulphate, (C) sodium citrate, (D) sodium sulphate, and (E) potassium sulphate. The solvents ethanol and n‐hexane were kept constant for all systems

In order to design a basic TLPSOES consisted of n‐hexane, ethanol and salt, the different salts were firstly investigated, including ammonium citrate, ammonium sulphate, sodium citrate, sodium sulphate, and potassium sulphate. They showed different effects on the formation of TLPSOESs, separation of EPA‐ & DHA‐rich oils, and other components. Among the tested salts, ammonium citrate and ammonium sulphate achieved insignificantly higher extraction efficiency of KO (*P* < 0.05), i.e. 91.85% (Figure 2A) and 91.81% (Figure 2B), than sodium citrate (83.78%, Figure 2C), sodium sulphate (90.24%, Figure 2D), and potassium sulphate (89.64%, Figure 2E), respectively, as well as extraction of DHA and EPA. Moreover, ammonium citrate makes a clear and transparent TLPSOES with top n‐hexane phase, small middle ethanol phase, bottom aqueous phase, and the solid interface between middle and bottom phase. This kind of salt (ammonium citrate) was also used to extract phenolic compounds from grape seeds using a microwave‐assisted aqueous two‐phase extraction system (MAATPES) [29]. Different salts such as NaCl, KCl, Na_2_SO_4_, KH_2_PO_4_, K_2_CO_3_, NaH_2_PO_4_, K_2_HPO_4_, and Na_3_PO_4_ have been reported for salting‐out extraction of bio‐based chemicals like 1,3‐propanediol and 2,3‐butanediol, etc. At the same time, the recovery yield was affected by temperature, pH, solvent and salt concentration [23]. The ammonium sulphate concentration in the range of 20–50% has been reported in salting‐out extraction of oil from microalgae and natural products [24].

#### 3.2.2 Solvents

Various solvents (ethanol, n‐hexane, acetone, n‐butanol, and n‐propanol) were used to design a suitable TLPSOESs with different concentration ranges, when ammonium citrate was kept constant for all solvents. Among the different solvents, ethanol and n‐hexane formed a clear TLPSOES, while acetone and n‐hexane did not form a TLPSOES, neither n‐butanol and n‐propanol. As shown in Figure 2A, the TLPSOES composed of ammonium citrate/ethanol/n‐hexane achieved the highest extraction efficiency of KO, EPA, and DHA in the top phase, the highest amount of protein in the solid interface between the middle and bottom phase, and the major part of flavonoids in the middle ethanol phase of TLPSOES. The remaining small amounts of KO, soluble proteins and other minor components are collected in the middle ethanol‐rich phase. This TLPSOES was chosen for further experimental evaluation.

### 3.3 Evaluation of TLPSOES using response surface methodology (RSM)

The BBD was generated using RSM to optimize the parameters of TLPSOES. The brief experimental correlation and data are given in Tables 1 and 2. The significant factors shown in Table 1, i.e. ammonium citrate, stirring time, ethanol, and n‐hexane, should be optimized moreover. The correlated relationship of every independent factor was investigated using 2nd order polynomial function to the responses in all phases in TLPSOES. The extraction efficiency of KO, EPA, DHA, proteins, and flavonoids was determined to each design to find out the regions with the highest efficiency, as given in Table 2. The response surface methodology was applied to optimize the significant variables affecting the DHA production by microalgae, including glucose, yeast extract, NaCl, pH, and incubation time [12, 27].

**TABLE 2:** The Box–Behnken Design original experimental form of significant variables and corresponding results of an extraction efficiency of KO, EPA, DHA, proteins, and flavonoids

					Extraction efficiency (%)				
Run Ord.	A	B	C	D	KO	EPA	DHA	Proteins	Flavonoids
01	20	30	25.0	42.5	84.42	83.33	82.25	80.62	78.23
02	10	30	17.5	42.5	81.05	80.17	80.38	79.36	78.01
03	10	80	25.0	42.5	85.92	83.34	84.21	81.78	79.01
04	10	130	17.5	42.5	87.81	87.39	86.86	83.67	79.12
05	20	130	17.5	25.0	70.69	69.41	68.11	85.98	77.34
06	20	80	17.5	42.5	90.66	90.13	89.14	88.11	79.29
07	30	80	25.0	42.5	88.11	86.15	85.78	84.77	78.93
08	30	80	17.5	25.0	68.26	66.62	67.44	86.89	79.19
09	10	80	10.0	42.5	86.32	84.79	85.15	83.64	61.03
10	30	130	17.5	42.5	90.35	90.04	90.13	88.24	75.84
11	30	30	17.5	42.5	79.25	78.54	76.55	81.37	72.65
12	10	80	17.5	25.0	66.57	66.32	65.16	83.49	73.35
13	20	30	17.5	25.0	67.77	66.81	66.57	84.78	75.26
14	20	80	17.5	42.5	90.64	90.13	89.11	88.13	79.24
15	20	80	17.5	42.5	90.65	90.14	89.12	88.16	79.22
16	20	80	17.5	42.5	90.66	90.13	89.15	88.12	79.28
17	20	30	17.5	60.0	83.44	82.21	81.13	85.29	79.31
18	20	30	10.0	42.5	80.25	79.23	79.92	82.28	60.89
19	20	80	10.0	60.0	90.13	90.01	88.96	87.34	69.45
20	10	80	17.5	60.0	89.22	88.18	88.06	85.54	74.68
21	30	80	17.5	60.0	90.67	90.45	90.08	88.19	78.93
22	20	130	25.0	42.5	90.89	90.37	90.44	85.34	79.31
23	20	80	17.5	42.5	90.63	90.15	89.07	88.10	79.28
24	20	80	17.5	42.5	90.67	90.12	89.16	88.13	79.27
25	20	80	25.0	60.0	90.68	90.45	90.27	85.58	79.32
26	20	130	17.5	60.0	90.95	90.49	90.63	86.32	79.22
27	20	80	25.0	25.0	67.65	65.28	66.28	85.99	79.02
28	20	80	10.0	25.0	64.23	64.08	64.03	86.84	56.95
29	20	130	10.0	42.5	89.93	89.11	88.87	87.67	60.86
30	30	80	10.0	42.5	88.14	87.09	87.48	88.03	59.64

A, ammonium citrate; B, stirring time; C, ethanol; D, n‐hexane.

The quadratic equations were generated against responses correlation to significant factors as mentioned below:

$$\begin{array}{ccc} E_{\mathrm{KO}} & = & -28.4+0.810A+0.1994B+1.525C+3.389D\\ & & -\;0.02313A^{2}-0.001169B^{2}-0.0300C^{2}\\ & & -\;0.03262D^{2}+0.00217A*B+0.0012A*C\\ & & -\;0.00034A*D-0.00214B*C+0.001311B*D\\ & & -\;0.00547C*D \end{array}$$

$$\begin{array}{ccc} E_{\mathrm{EPA}} & = & -26.2+0.822A+0.1860B+1.572C+3.255D\\ & & -\;0.02676A^{2}-0.001169B^{2}-0.0383C^{2}\\ & & -\;0.03273D^{2}+0.00214A*B+0.0017A*C\\ & & +\;0.00281A*D-0.00189B*C+0.00162B*D\\ & & -\;0.00145C*D \end{array}$$

$$\begin{array}{ccc} E_{\mathrm{DHA}} & = & -17.2+0.716A+0.1053B+1.089C+3.192D\\ & & -\;0.02190A^{2}-0.001148B^{2}-0.0248C^{2}\\ & & -\;0.03187D^{2}+0.00355A*B-0.0025A*C\\ & & -\;0.00037A*D-0.00051B*C+0.002274B*D\\ & & -\;0.00179C*D \end{array}$$

$$\begin{array}{ccc} E_{\mathrm{Proteins}} & = & 56.78+1.051A+0.1935B+1.028C+0.074D\\ & & -\;0.02150A^{2}-0.001062B^{2}-0.02730C^{2}\\ & & +\;0.00003D^{2}+0.001280A*B-0.00467A*C\\ & & -\;0.00107A*D-0.00045B*C\\ & & -\;0.000049B*D-0.00173C*D \end{array}$$

$$\begin{array}{ccc} E_{\mathrm{Flavonoids}} & = & -19.3+0.696A+0.0810B+6.902C\\ & & +\;0.719D-0.01898A^{2}-0.000476B^{2}\\ & & -\;0.1398C^{2}0.00145D^{2}+0.00104A*B\\ & & +\;0.0044A*C-0.00227A*D\\ & & +\;0.00074B*C-0.00062B*D\\ & & -\;0.02324C*D \end{array}$$

where E is the possible extraction efficiency of responses such as KO, EPA, DHA, proteins and flavonoids. The A, B, C, and D denote the factors, i.e. ammonium citrate, stirring time, ethanol, and n‐hexane, respectively. Table 3 shows the regression coefficients significance of each equation obtained by Analysis of Variance (ANOVA).

**TABLE 3:** The ANOVA (Analysis of variance) for all quadratic equation of responses extraction efficiency

Responses	*R* ^2^	*R* ^2^ (Adj.)	*R* ^2^ (Pred.)	SD (%)	Model F‐value	Model *P*‐value
*E*_KO_	0.9815	0.9642	0.8934	1.700	56.82	<0.0001
*E*_EPA_	0.9799	0.9799	0.9612	1.800	52.35	<0.0001
*E*_DHA_	0.9814	0.9814	0.9639	1.722	56.38	<0.0001
*E*_Proteins_	0.9224	0.9224	0.8500	1.808	12.73	<0.0001
*F*_Flavonoids_	0.9540	0.9110	0.7348	1.700	22.20	<0.0001

The maximum F‐value (56.82) was correlated with a lower *P*‐value (0.001) for the model, which proved that the model was valid (Table 4). As indicated in Table 4, the *P*‐values lower than 0.001 predict the high significance of the models. In all phases of TLPSOES, the extraction efficiency was affected by independent factors showing a significant difference (*P* < 0.05). Moreover, the model different values recommended a better association among results and model. The higher value of lack of fit (43.78) suggested that the model predicts the extraction efficiency with various correlations of the factors’ values. These facts show that the model predicts a high degree of precision and better reliability of the experiments. The model could navigate the design space, while its equation was adequate, credible, and reproducible for predicting the KO, EPA, DHA, proteins, and flavonoids extraction efficiency rate under integration of values of variables.

**TABLE 4:** The statistical parameters of the model and ANOVA analysis of KO

Source	DF	MS	SS	F‐value	*P*‐value
Model	14	164.33	2300.63	56.82	0.000
A	1	5.19	5.19	1.79	0.200
B	1	164.58	164.58	56.90	0.000
C	1	6.26	6.26	2.17	0.162
D	1	1406.60	1406.60	486.32	0.000
A^2^	1	36.68	36.68	12.68	0.003
B^2^	1	58.53	58.53	20.24	0.000
C^2^	1	19.54	19.54	6.75	0.020
D^2^	1	684.23	684.23	236.57	0.000
AB	1	4.71	4.71	1.63	0.221
AC	1	0.03	0.03	0.01	0.915
AD	1	0.01	0.01	0.00	0.945
BC	1	2.58	2.58	0.89	0.360
BD	1	5.27	5.27	1.82	0.197
CD	1	2.06	2.06	0.71	0.412
Error	15	2.89	43.39		
Lack of fit	10	4.34	43.38	0.93	0.000
Pure error	5	0.00	0.00		

**FIGURE 3:**
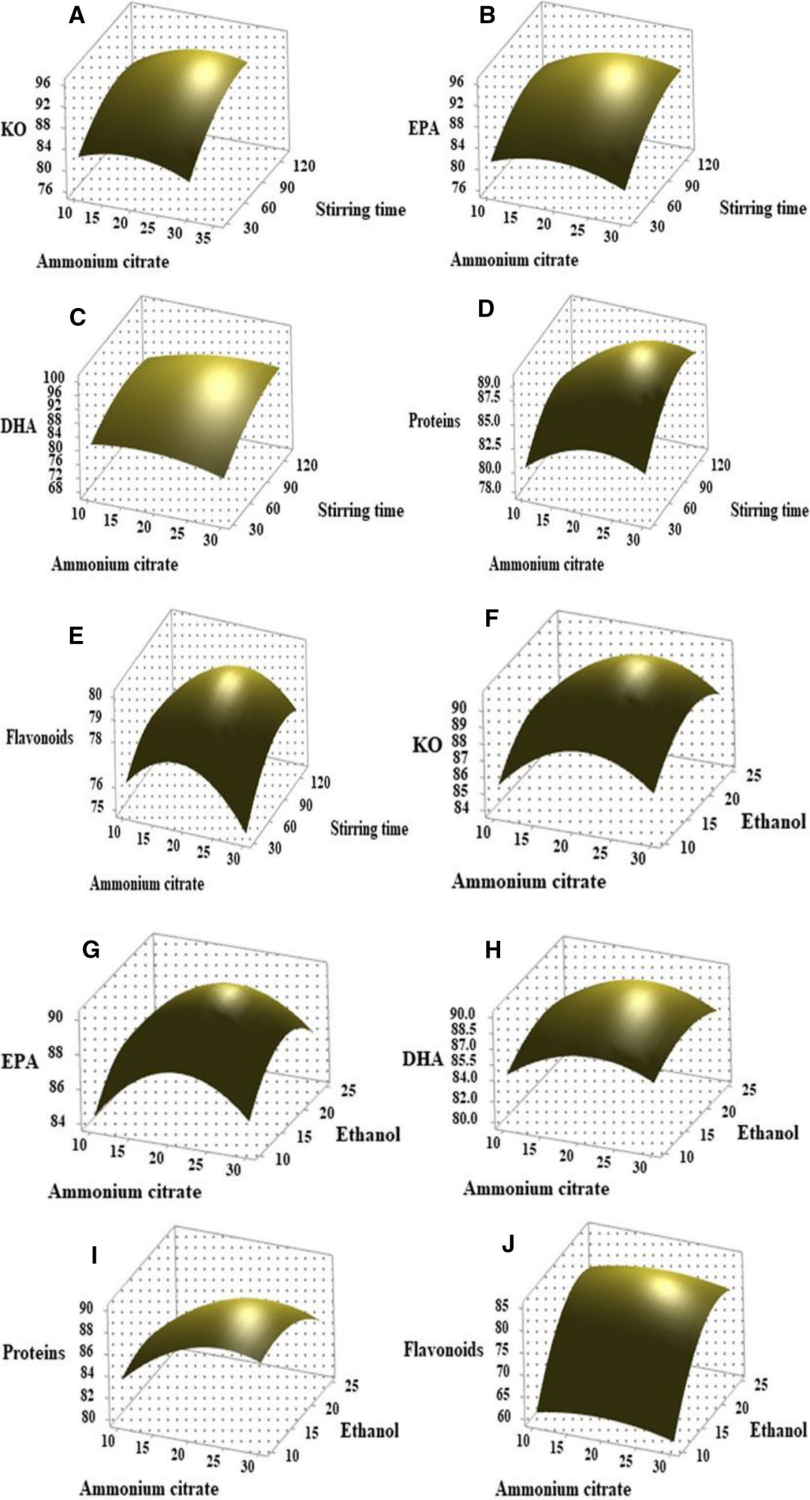
The effects of significant variables (ammonium citrate, stirring time, and ethanol) on the BBD response surface curves against extraction efficiency of KO (A, F), EPA (B, G), DHA (C, H), flavonoids (E, J), and proteins (D, I)

**FIGURE 4:**
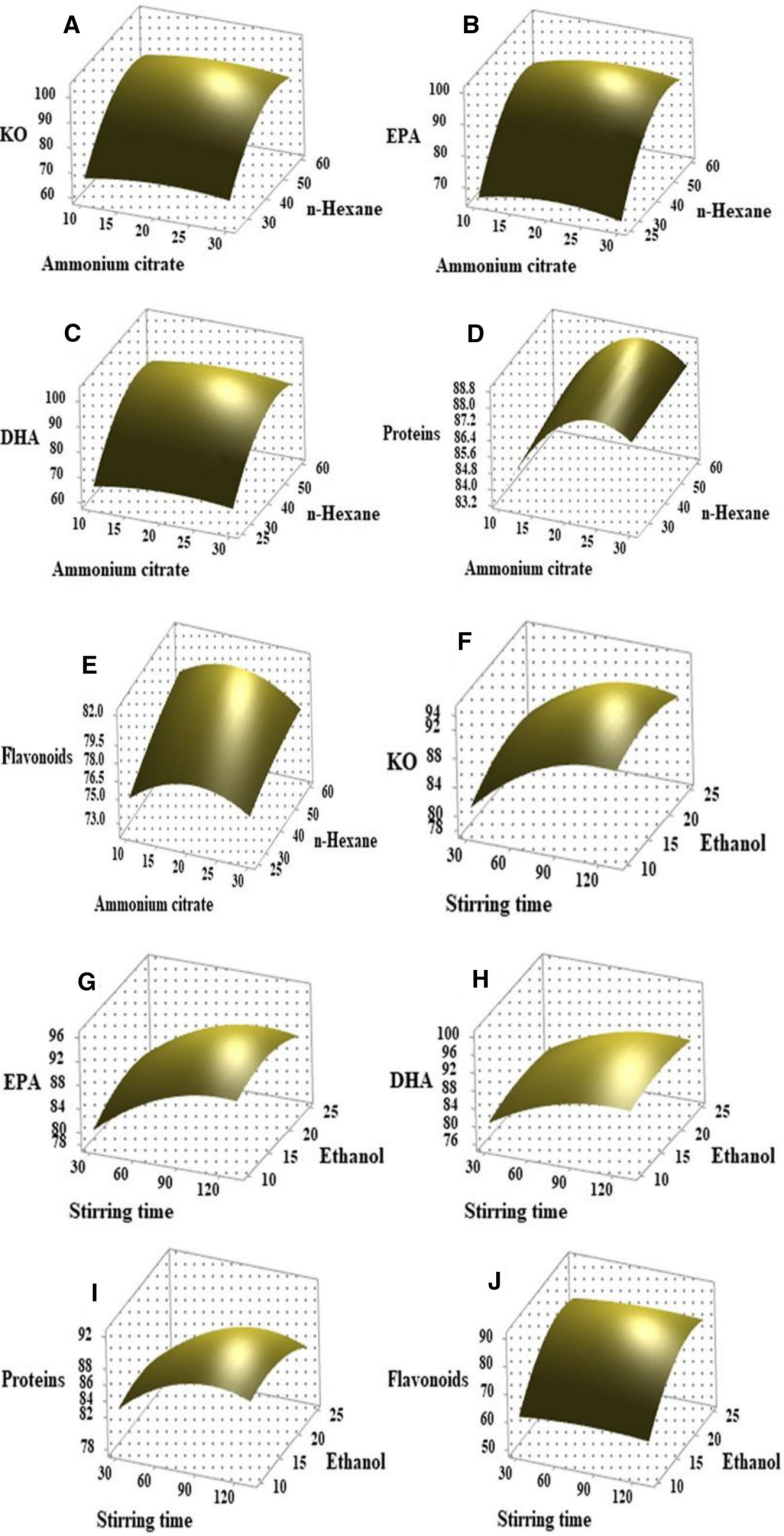
The effects of significant variables (ammonium citrate, stirring time, n‐hexane, and ethanol) on the BBD response surface curves against extraction efficiency of KO (A, F), EPA (B, G), DHA (C, H), flavonoids (E, J), and proteins (D, I)

**FIGURE 5:**
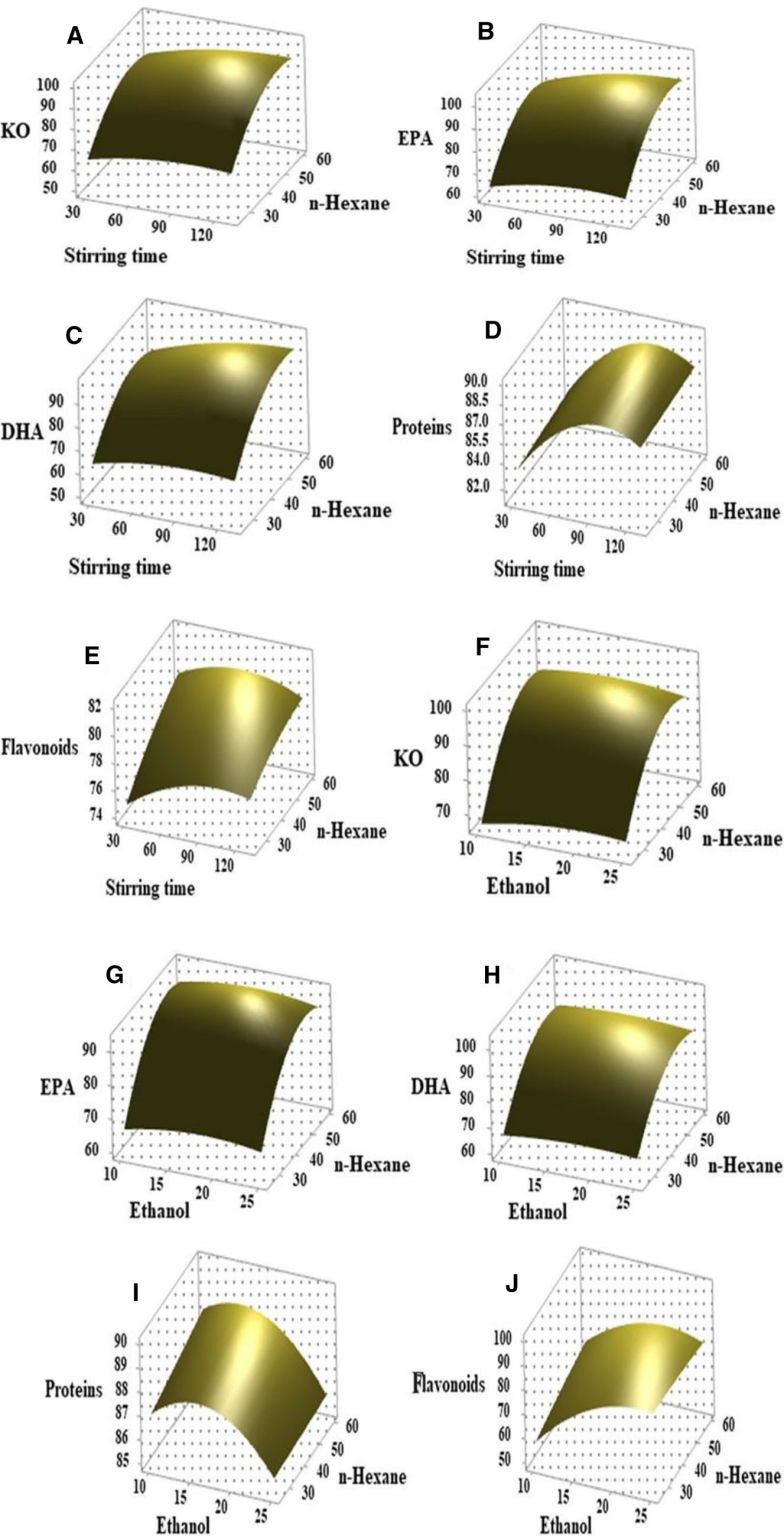
The effects of significant variables (stirring time, n‐hexane, and ethanol) on the BBD response surface curves against extraction efficiency of KO (A, F), EPA (B, G), DHA (C, H), flavonoids (E, J), and proteins (D, I)

To observe three‐dimensional response surface curves within experimental limit and maximize the extraction efficiency, every two variables were changed. The associated effects of ammonium citrate/stirring time, ammonium citrate/n‐hexane, ammonium citrate/ethanol, stirring time/n‐hexane, stirring time/ethanol, and n‐hexane/ethanol were investigated on the extraction efficiency of KO, EPA, DHA, proteins, and flavonoids (Figures 3, 4, 5). As shown in Figure 3A‐J, the different response curves of extraction efficiency of KO, EP, DHA, proteins, and flavonoids were affected by ammonium citrate, stirring speed time and ethanol. When the krill was treated by a TLPSOES of 20% ammonium citrate, 17.5% ethanol, and 42.5% n‐hexane for stirring time of 80 min, the extraction efficiency KO, EP, DHA, proteins, and flavonoids was 90.66%, 90.13%, 89.15%, 88.12%, and 79.28%, respectively (Table 2). The increase in stirring time duration tends to incline the separation of KO, EP, and DHA in top *n*‐hexane‐rich phase (Figure 3). While the decrease in n‐hexane concentration tends to decline the KO, EP, and DHA in top *n*‐hexane‐rich phase (Figure 4). The highest extraction efficiency of KO, EPA, DHA, proteins, and flavonoids was attained at 20% w/w of ammonium citrate, 80 min of stirring time, and 42.5% w/w of n‐hexane (Table 2 and Figures 3, 4, 5).

As shown in Figures 3, 4, 5, the responses curves to extraction efficiency were different for each component. The responses of extraction efficiency of KO, EPA, DHA, proteins, and flavonoids change against variation in ammonium citrate, stirring time, ethanol, and n‐hexane (Figure 4A‐J). The associated effects of ammonium citrate, stirring time, ethanol, and n‐hexane on bioactive component's extraction efficiency were reported in Figure 4.

As shown in Figures 3–5A‐J, the upper convex shapes of response surfaces with the maximum points in the experimental domains indicated that the ranges of variables chosen were reasonable. At optimum concentration of ammonium citrate (20%), ethanol (17%), n‐hexane (42.5%), and stirring time (80 min), the highest extraction efficiency (above 80%) of KO, EPA, DHA, and proteins was attained respectively. While at same parameters the extraction efficiency of flavonoids was below 80% (Table 2). All variables in this study show a significant effect on the separation of KO, EPA, DHA, proteins and flavonoids in TLPSOESs (Figure 3, 4, 5). Therefore, the independent parameters attained the maximum extraction efficiency of bioactive components recommend the optimum parameters for various aims of separation. The correlated effects of each two variables, their optimum range, and responses can be seen from Figure 3, 4, 5. For the convenience of practical experiment, the variables range would be determined further. The KO was extracted using subcritical dimethyl ether while the parameters were optimized by Box–Behnken response surface design [12].

### 3.4 Optimization of significant parameters for TLPSOES

**FIGURE 6:**
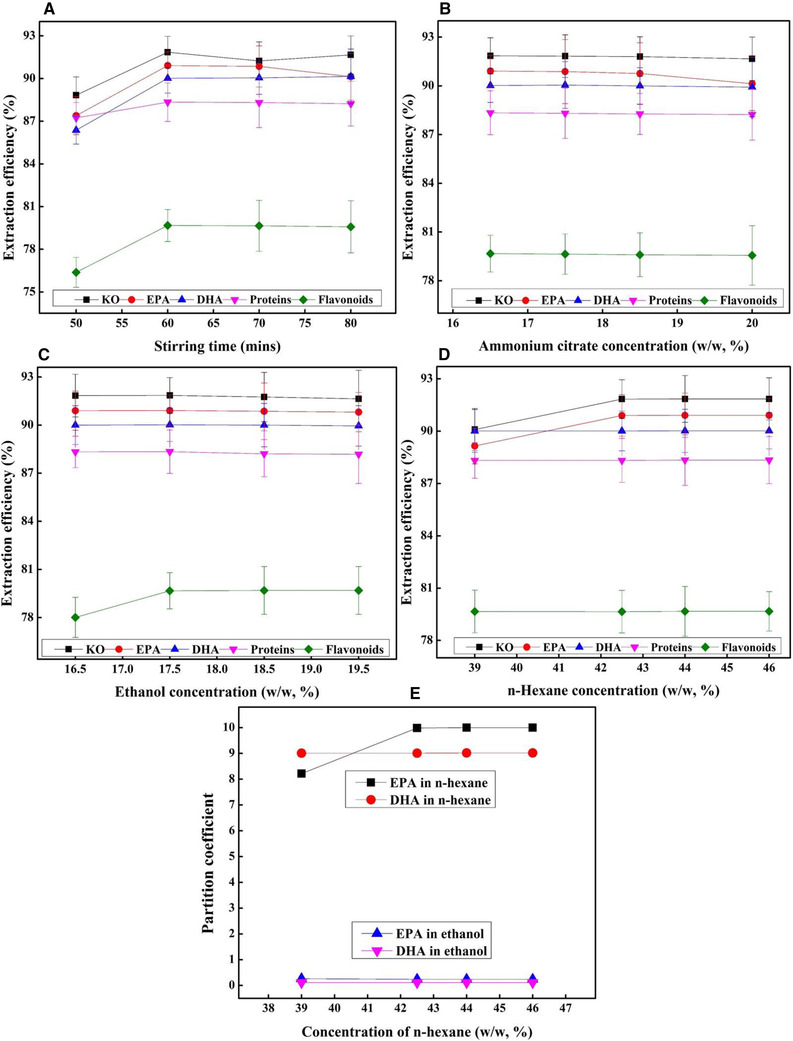
The effects of (A) stirring time (B) ammonium sulphate (C) ethanol, and (D) n‐hexane on the extraction efficiency of KO, EPA, DHA, proteins, and flavonoids in a TLPSOES. (E) Effect of n‐hexane concentration (39–46%) on the partition behavior of EPA and DHA

On the basis of economic and extraction efficiency, a set of parallel experiments were performed for ammonium citrate, stirring time, ethanol, and n‐hexane to determine the optimum TLPSOES parameters. The lowest possible range of ammonium citrate (16.5%, w/w) was selected to utilize minimum salt concentration and obtain the highest extraction efficiency. At this concentration, the extraction efficiency of EPA, DHA, and KO was 90.91 ± 0.97%, 90.02 ± 1.04%, and 91.85 ± 1.11%, respectively, as indicated in Figure 6B. These extraction efficiencies were not affected much in a close concentration range of ammonium citrate from 16.5 to 19% as shown in Figure 6B. The high and low ammonium citrate concentration influences the extraction efficiency of proteins, EPA, DHA, oils, and flavonoids shown in Figures 3 and 4. The stirring time was also a key factor to affect the extraction efficiency of KO, EPA, DHA, proteins and flavonoids in TLPSOES. As shown in Figure 6A, when the stirring time duration was 70 min, the extraction efficiency of KO, EPA, DHA, proteins and flavonoids was 91.85, 90.91, 90.02, 88.34, and 79.67%, respectively. Similarly, when increasing the duration, the same extraction efficiency was reported with no dramatic changes (Figure 6A). Therefore, the minimum stirring time duration was chosen for optimized TLPSOES. The stirring extraction time reported previously to extract KO was 90 min, while our study reduced the time to 70 min [2, 12].

Both ethanol and n‐hexane concentrations were investigated in TLPSOES to separate proteins, flavonoids, and DHA‐ and EPA‐rich KO. As shown in Figure 6D, when ethanol concentration was kept smaller (17.5%) compared to n‐hexane (46%), the EPA and DHA extraction efficiency was 90.91% and 90.02% in the top n‐hexane phase. It has been reported that 40% w/w of n‐hexane recovered the highest oil yield from *Schizochytrium limacinium* SR21 using three‐phase partitioning system [24]. Another study using 28% of n‐hexane in an TLPSOE recovered 86.70% of oils from waste liquor processing sea cucumber [19].

Figure 6C showed the effect of ethanol concentration on the extraction efficiency of flavonoids, in which more flavonoids were separated in the ethanol‐rich phase at 17.5%. Although the extraction efficiency of flavonoids increased as ethanol concentration increased from 17.5% to 20%, the extraction efficiency of KO in the n‐hexane‐rich phase decreased. When the ethanol concentration was low, e.g. less than 17.5%, the flavonoids content also reduced because they dissolve partly in the n‐hexane‐rich phase (Figure 6C). The smaller ethanol concentration could benefit to dissolve a remaining small amount of soluble proteins, oils, and flavonoids in TLPSOES. The EPA and DHA partition coefficient increased when the n‐hexane concentration increased above 39% and didn't show a big difference until 46% (Figure 6E).

### 3.5 Removal of fluoride by TLPSOES

The fluoride content in the whole wet krill was determined to be 324 ± 2.22 mg/kg using IC as indicated in Figure 1B. As reported previously, the fluoride concentration in krill is different in various parts of the body and origin of sources [5, 31, 32]. Furthermore, it depends on the post‐mortem migration and residual cuticle of fluoride [32], as well as fishing season and sea area, pretreatment mode, freezing temperature and time, drying or not [33].

Although conventional extraction could decrease the fluoride content in KO to 33.67 ± 1.78 mg/kg, further purification would still be needed to remove the higher fluoride amount. The absorbent or chelating agent and n‐hexane were added to KO, stirred at room temperature for 150 min, centrifuged, and stored. A similar procedure will be repeated until the concentration of fluoride gets to the optimum range.

**FIGURE 7:**
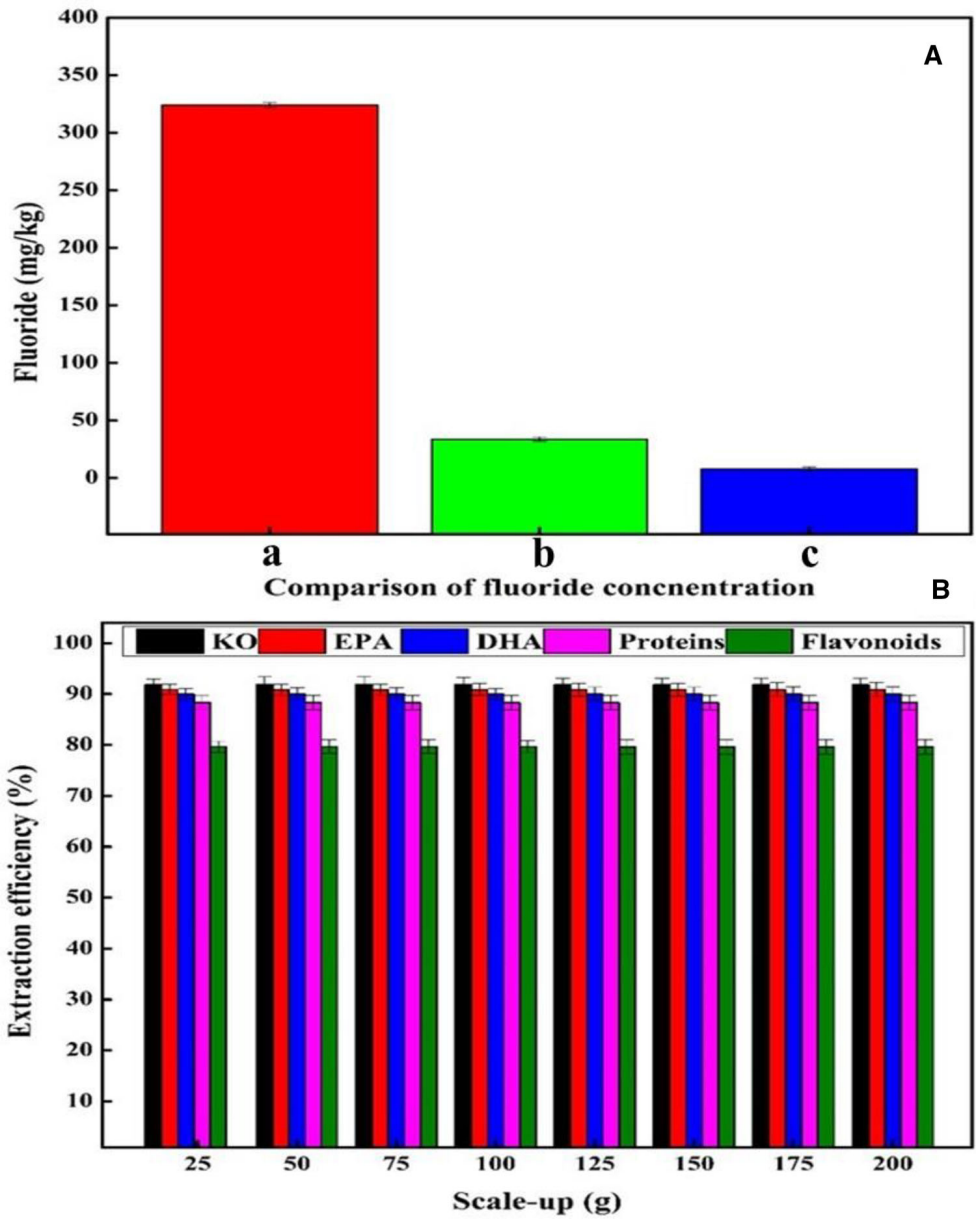
(A) Comparison of fluoride concentration in (a) whole wet krill (b) KO extracted by a conventional method, and (c) KO extracted by TLPSOES. (B) Effects of various scale‐up on the extraction efficiency of KO, EPA, DHA, proteins, and flavonoids in TLPSOES using wet krill biomass. The 25, 50, 75, 100, 125, 150, 175, 200 g shows the TLPSOES total composition

Compared to conventional solvent approach, the KO extracted by TLPSOES contains the optimum range of fluoride concentration (7.82 ± 1.69 mg/kg) as indicated in Figure 7A. Therefore, it would not need further purification steps, which ultimately reduce cost and time. It has been reported that ingestion of 4 mg fluoride per day is beneficial for bone structure maintenance and tooth decay prevention [34]. Low fluoride level can be considered as a potential product for antioxidant and nutraceutical industry [35].

### 3.6 TLPSOES scale‐up at laboratory

The feasibility of TLPSOES for wet krill biomass was investigated at laboratory level up to 200 g under the optimum conditions. The optimum conditions of TLPSOES were 16.5% w/w of ammonium citrate, 17.5% w/w of ethanol, and 46% w/w of n‐hexane. Figure 7B indicated that TLPSOES obtained the highest extraction efficiency for EPA, DHA, KO, flavonoids and proteins. The highest extraction efficiency of these components was constant when the system was gradually enlarged up to 200 g. A slight difference in the recovery yield was recorded when the system was 175–200 g. This is similar to the previous reports about TLPSOES scale‐up from 20 to 60 g [15] or from 10 g to 5 kg [22], and reveals that TLPSOES could be an effective method to separate bioactive components from wet krill biomass on a large scale.

## 4 CONCLUDING REMARKS

A higher concentration of fluoride in krill restricting its utilization for human use. A novel TLPSOES approach has been applied to separate multiple valuable components and KO with an optimum range of fluoride simultaneously in the same system. The parameters of TLPSOES were optimized by BBD using RSM for KO extraction efficiency. Under the optimized conditions, TLPSOES attained maximum extraction efficiency of 90.91 ± 0.97% EPA, 90.02 ± 1.04% DHA, 91.85 ± 1.11% KO, 88.34 ± 1.35% proteins, and 79.67 ± 1.13% flavonoids, respectively. The laboratory scaled‐up was conducted for optimized TLPSOES from 22 to 200 g. Compared to whole wet krill and oils extracted by conventional method, the TLPSOES has prominent advantages of lowest fluoride level with multiple components separation. The TLPSOES extraction procedure could be employed in food processing and separation areas to extract organic constituents in the future.

## CONFLICT OF INTEREST

The authors have declared no conflict of interest.

## Data Availability

The data that support the findings of this study are available from the corresponding author upon reasonable request.
